# Pulmonary Sequestration: Early Diagnosis and Management

**DOI:** 10.1155/2015/454860

**Published:** 2015-07-26

**Authors:** Sajad A. Wani, Gowher N. Mufti, Nisar A. Bhat, Ajaz A. Baba

**Affiliations:** Department of Paediatric Surgery, SKIMS, Soura, Srinagar, Jammu and Kashmir 190011, India

## Abstract

Intralobar sequestration is characterized by aberrant formation of nonfunctional lung tissue that has no communication with the bronchial tree and receives systemic arterial blood supply. Failure of earlier diagnosis can lead to recurrent pneumonia, failure to thrive, multiple hospital admissions, and more morbidity. The aim of this case report is to increase the awareness about the lung sequestration, to diagnose and treat it early, so that it is resected before repeated infection, and prevent the morbidity and mortality.

## 1. Introduction

Pulmonary sequestration also known as accessory lung is a cystic or solid mass of nonfunctioning primitive segmental lung tissue that does not communicate with the tracheobronchial tree and has anomalous systemic blood supply. It is a bronchopulmonary foregut malformation with estimated incidence of 0.1 to 6.4% [1]. Anatomically, it is classified into intralobar sequestration (ILS) and extralobar sequestration (ELS) based on the relationship of the aberrant segmental lung tissue to the pleura. Intralobar sequestration is more common and accounts for the majority (75–85%) of all sequestration [2, 3]. We presented a case of intralobar sequestration to increase the awareness about the condition and its earlier diagnosis and management.

## 2. Case Report

**Figure 1:**
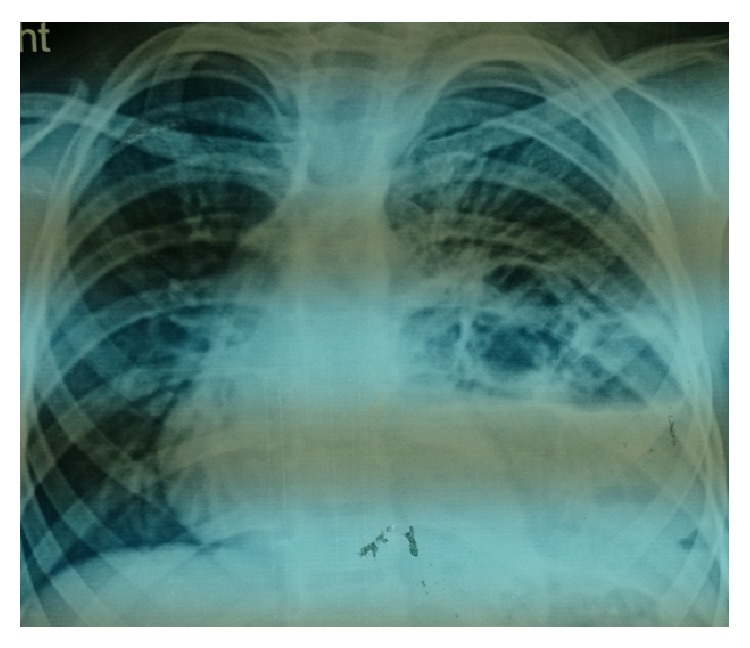
X-chest showing an opacity in the left lower lobe of the lung.

**Figure 2:**
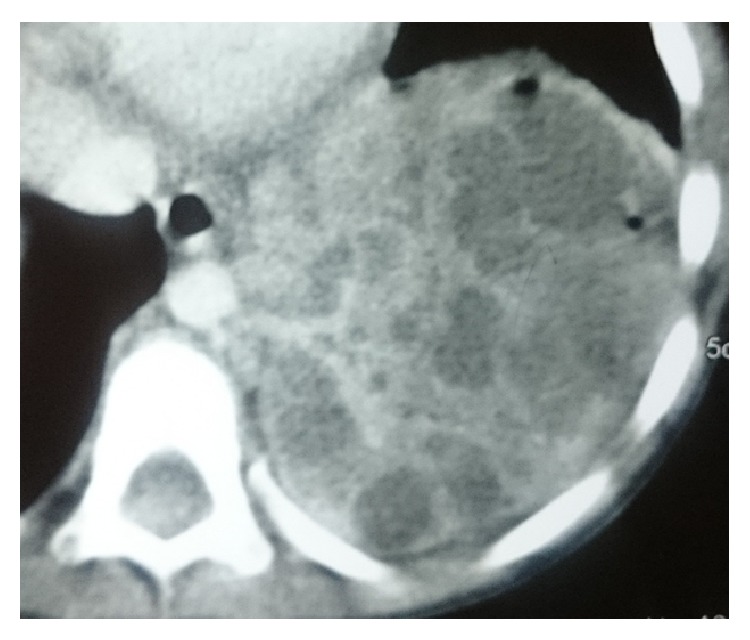
Computed tomography (CT) of chest revealed a multiloculated mass in the left lower lobe of the lung.

**Figure 3:**
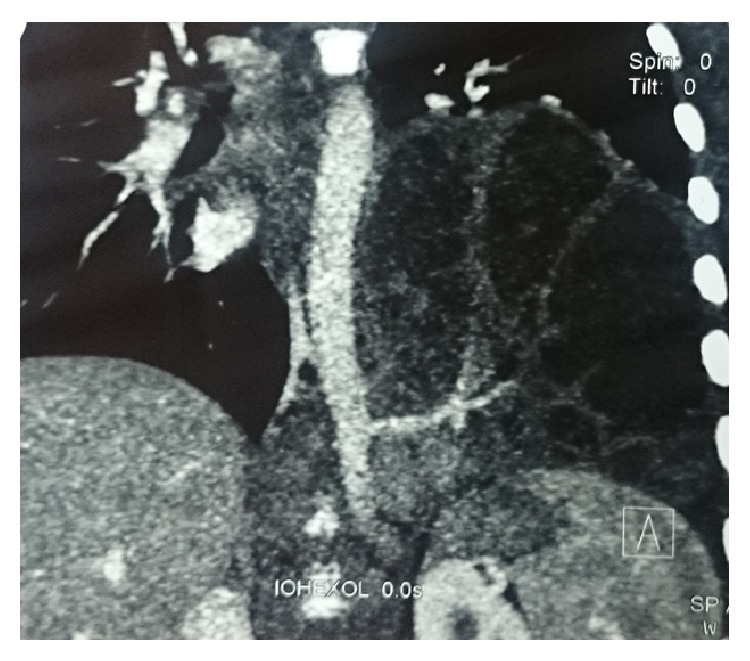
CT angiography shows intralobar lung sequestration with anomalous blood supply from the descending thoracic aorta.

Two year male child was admitted to our department with history of cough and fever with X-chest showing an opacity in the left lower lobe of the lung (Figure 1). In the past, he was admitted thrice to the district hospital as a case of pneumonia. Computed tomography (CT) of chest revealed a multiloculated mass in the left lower lobe of the lung, consistent with sequestration versus possible congenital pulmonary airway malformation (Figure 2). CT angiography was done which shows intralobar lung sequestration with anomalous blood supply from the descending thoracic aorta and venous drainage via pulmonary vein (Figure 3). He was operated on, anomalous feeding vessel was ligated first, and then left lobectomy was done. Postoperative period was uneventful and the patient was discharged on 4th postoperative day and he is doing well in followup. Histopathological examination confirmed the diagnosis of intralobar lung sequestration.

## 3. Discussion

Felker and Tonkin described the lung sequestration as a malformation that is comprised of dysplastic lung tissue with no tracheobronchial tree communication and that receives anomalous systemic arterial supply [4]. Intralobar sequestration (ILS) is surrounded by normal lung tissue and pleura while extralobar sequestration (ELS) has its own pleural investment. The age of presentation is dependent on the type of sequestration and this in turn determines the clinical presentation. ILS is four times more common than ELS. Intralobar sequestration presents late in childhood or adolescence with recurrent pulmonary infection while extralobar sequestration more commonly presents in newborn with respiratory distress, cyanosis, and infection.

The most common location of ILS is in the posterior basal segments of the lung and nearly two third appearing in the left lung. Associated congenital anomalies are uncommon in ILS. In intralobar sequestration, anomalous systemic arterial supply is via the descending thoracic aorta (72%), as seen in our case, via abdominal aorta, celiac axis, or splenic artery (21%), via intercostal artery (3%), and rarely via the subclavian, internal thoracic, and pericardiacophrenic arteries. Venous drainage is usually via the pulmonary veins, but it can also occur through the azygos vein/hemiazygos system, portal vein, right atrium, or IVC.

Chest radiograph can provide a diagnostic clue to lung sequestration. In a patient with recurrent localized pulmonary infection with opacity in the X-ray chest as in our patient, it is suggestive of lung sequestration. In the past, aortography was frequently used for diagnosis. However, the gold standard for identifying the pulmonary sequestration recently is CT/MR angiography as it confirms the anatomy, identifies the anomalous systemic arterial supply, and shows the venous drainage [5].

Management of the asymptomatic lung sequestration is controversial. However, most authors advocate resection of these lesions because of the likelihood of recurrent infection, the need for larger resection if the sequestration becomes chronically infected, and the possibility of hemorrhage [6]. Surgical resection is the treatment of choice and ILS often requires lobectomy. Open thoracotomy remains the best approach with safe isolation and division of anomalous systemic feeding arteries. However, complete thoracoscopic resection has been reported with low morbidity and mortality [7].

## 4. Conclusion

Congenital pulmonary sequestration is a rare lung malformation. In any patient with localized recurrent pulmonary infection, lung malformation should be suspected. These patients should be referred to tertiary care center where they can be diagnosed and treated early, so as to prevent the morbidity and mortality associated with late resection of the lesion.
